# Association between ambulatory blood pressure monitoring parameters and left ventricular hypertrophy in an Ecuadorian population

**DOI:** 10.3389/fmed.2026.1767168

**Published:** 2026-02-16

**Authors:** Patricia Delgado Cedeño, José Espinoza-Plaza, Estefanía Arteaga Herrera, Joao Tumbaco Mite, Killen H. Briones Zamora, Killen H. Briones Claudett

**Affiliations:** 1 Instituto Ecuatoriano del Corazón (IECOREC), Guayaquil, Ecuador; 2 Universidad de Especialidades Espiritu Santo, Samborondon, Ecuador; 3 Universidad Internacional del Ecuador (UIDE), Quito, Ecuador; 4 Briones PulmoCare, Guayaquil, Ecuador

**Keywords:** ambulatory blood pressure monitoring, diagnostic accuracy, Ecuador, hypertension, Latin America, left ventricular hypertrophy, ROC analysis

## Abstract

**Background:**

Systemic arterial hypertension (SAH) is the leading modifiable cardiovascular risk factor worldwide and a major cause of hypertension-mediated organ damage (HMOD), including left ventricular hypertrophy (LVH). Ambulatory blood pressure monitoring (ABPM) provides a more accurate assessment of hemodynamic load and circadian blood pressure variability than office measurements. However, its diagnostic performance for LVH detection has not been well studied in Latin American populations.

**Objective:**

To evaluate the cross-sectional diagnostic discrimination of ABPM-derived blood pressure parameters for detecting echocardiographic LVH in an Ecuadorian hypertensive cohort.

**Methods:**

We conducted a cross-sectional study including 110 adults who underwent 24-h ABPM and three-dimensional echocardiography from October 2021 to June 2022 at the Instituto Ecuatoriano del Corazón (IECOREC), Guayaquil, Ecuador. LVH was defined using sex-specific left ventricular mass index thresholds (>115 g/m^2^ men; >95 g/m^2^ women). Receiver operating characteristic (ROC) curves were generated for ABPM parameters, and optimal cut-off values were determined using Youden’s index. All cut-offs are presented as exploratory and sample-dependent, requiring external validation.

**Results:**

LVH was present in 34.5% (*n* = 38) of participants. Median systolic blood pressure (SBP) values were higher in the LVH group across all periods: 24-h SBP (128 vs. 125 mmHg; *p* = 0.038), daytime SBP (133 vs. 130 mmHg; *p* = 0.043), and nighttime SBP (119 vs. 115 mmHg; *p* = 0.011). ROC analysis showed modest discriminative performance for all ABPM parameters (all AUCs <0.65). Daytime SBP demonstrated the best diagnostic balance (AUC = 0.620; 95% CI: 0.523–0.717; *p* = 0.047) with an optimal cut-off of ≥134 mmHg (sensitivity 45.0%, specificity 77.8%, Youden’s J = 0.228). Nighttime diastolic load >30% showed high sensitivity (89.5%) but low specificity (29.2%; Youden’s J = 0.19), suggesting potential utility for triage rather than confirmation. Diastolic parameters showed poor discrimination (AUCs 0.515–0.607).

**Conclusion:**

In this Ecuadorian cohort, ABPM-derived parameters showed modest cross-sectional discrimination for LVH detection, with daytime SBP demonstrating the best diagnostic balance. These findings are hypothesis-generating and do not support the use of individual ABPM parameters as stand-alone screening tools for LVH. Instead, ABPM should be regarded as a complementary tool for cardiovascular risk stratification and for prioritizing echocardiographic evaluation in resource-limited settings. The proposed thresholds are exploratory and require validation in larger, prospective, multicenter Latin American studies.

## Introduction

Systemic arterial hypertension (SAH) remains the leading modifiable cardiovascular risk factor worldwide and continues to be one of the major causes of morbidity and premature mortality in adults, accounting for approximately 10.8 million deaths annually (1). In Latin America, it is estimated that between 20 and 40% of adults suffer from hypertension, while in Ecuador, recent epidemiological data report prevalence rates close to 27–29% among the urban adult population, reflecting a critical public health burden (2, 3).

The clinical significance of SAH lies mainly in its potential to induce target organ damage—hypertension-mediated organ damage (HMOD)—which markedly increases the risk of cardiovascular events, renal failure, cognitive decline, and overall mortality (4). Among the earliest and most relevant manifestations of HMOD is left ventricular hypertrophy (LVH), an initial adaptive response of the myocardium to chronic pressure overload that becomes maladaptive over time, accelerating the development of heart failure, arrhythmias, and sudden cardiac death (4, 5). Imaging-confirmed LVH is associated with increased risk of major cardiovascular events, positioning it as a key target for prevention and treatment strategies in hypertension (5).

Although in-office blood pressure (BP) measurement remains the diagnostic cornerstone, this approach has critical limitations: it fails to capture circadian variability, is susceptible to white-coat and masked hypertension, and correlates poorly with subclinical organ damage (6). Consequently, 24-h ambulatory blood pressure monitoring (ABPM) is considered the reference method for characterizing hemodynamic load, enabling assessment of circadian patterns and quantification of pressure load, and is more informative for HMOD risk stratification (7).

Recent evidence suggests that nocturnal BP phenotypes and impaired dipping are strongly associated with LVH and cardiovascular risk, supporting the clinical relevance of nighttime systolic measures and related indices as integrated markers of chronic pressure exposure (8, 9).

Despite this international evidence, there is a paucity of studies evaluating the clinical utility of ABPM-derived systolic parameters—especially nighttime SBP and pressure load—in Latin American populations, where cardiovascular risk profiles and health-system constraints differ from the cohorts that underpin most guideline recommendations (3, 10). In Ecuador, local validation of ABPM-derived indices for LVH screening remains limited, despite restricted access to advanced cardiac imaging (10).

Accordingly, the objective of this study was to evaluate the cross-sectional diagnostic discrimination of selected ABPM-derived blood pressure indices for detecting echocardiographic LVH in a cohort of adult patients from Guayaquil, Ecuador. Our goal is to identify which ABPM-derived parameters are most informative for LVH detection and to provide locally relevant evidence that can guide cardiovascular risk stratification in settings with limited access to advanced imaging. Given the cross-sectional design, the present work is intended to quantify diagnostic discrimination at a single time point and should not be interpreted as causal or prognostic (11, 12).

## Materials and methods

### Study design, setting, and ethical approval

We conducted a cross-sectional, observational study with prospective recruitment at the Instituto Ecuatoriano del Corazón (IECOREC), Guayaquil, Ecuador. Participant enrollment and study procedures were conducted after ethics approval and occurred from October 2021 to June 2022.

The research protocol was reviewed and approved by the Institutional Bioethics Committee of the Universidad Técnica de Manabí (UTM), in accordance with Ecuadorian national regulations for research involving human subjects (Protocol No. UTM-2021-007; approval letter No. UTM II 2018-011-OF; session date: October 13, 2021). Written informed consent was obtained from all participants prior to inclusion, in accordance with the Declaration of Helsinki and national guidelines.

Additionally, the protocol was reviewed by the Ethics and Bioethics Committee of the Instituto Universitario Italiano de Rosario (IUNIR), Argentina, as part of doctoral program requirements.

Eligible participants were adults aged 18–79 years with a clinical indication for 24-h ambulatory blood pressure monitoring (ABPM), either for initial hypertension screening or follow-up within primary prevention. Participants were required to be capable of providing informed consent and have an adequate echocardiographic acoustic window for three-dimensional assessment. We excluded patients with prior extracardiac target organ damage (stroke, advanced chronic kidney disease with eGFR <30 mL/min/1.73m^2^), significant chronic liver disease (Child-Pugh class B or C), active alcohol or drug dependence, current malignancy or cancer history within the past 5 years, hypertensive disorders of pregnancy, Parkinson’s disease, significant cognitive impairment, or cardiac arrhythmias that could impair ABPM accuracy. These criteria were applied to enhance internal validity and measurement reliability; however, they may reduce representativeness, since multimorbidity (including arrhythmias and chronic kidney disease) is common among hypertensive adults in routine practice (13).

### Clinical and anthropometric assessment

A comprehensive medical history and physical exam were conducted. Collected data included demographics, cardiovascular risk factors (tobacco, alcohol, sedentary lifestyle, diet, occupational stress), comorbidities, and medication use. Anthropometric measures—body weight, height, waist circumference—were obtained with calibrated equipment following international protocols. Body mass index (BMI) was calculated as weight (kg) divided by height squared (m^2^). Office BP was measured after 5 min rest using an automated oscillometric device (Omron HEM-7120), with the average of 3 measurements recorded (14).

Waist circumference (WC) was measured at the midpoint between the lowest rib and the iliac crest during minimal respiration. Abdominal obesity categories were defined according to IDF/AHA/NHLBI harmonized criteria: normal (<94 cm men, <80 cm women), elevated risk (94–102 cm men, 80–88 cm women), and very high risk (>102 cm men, >88 cm women).

### Ambulatory blood pressure monitoring (ABPM)

ABPM utilized a validated device (Microlife WatchBP O3 – AFIB, model 3MZ1-1A) following European Society of Hypertension and regional recommendations (7, 10). BP readings were scheduled every 20 min from 06:00 to 22:00 and every 30 min from 22:00 to 06:00. Quality criteria were ≥70% valid readings, with a minimum of 20 daytime and 7 nighttime successful recordings; compliance was documented via patient diary (7).

Hypertension thresholds were defined as 24-h mean ≥130/80 mmHg, daytime ≥135/85 mmHg, and nighttime ≥120/70 mmHg, consistent with contemporary guideline cutoffs for ABPM interpretation (7, 13). Pressure load was defined as the percentage of readings exceeding the corresponding thresholds, with >50% classified as abnormal during the respective periods. Circadian patterns were classified as dippers (10–20% nocturnal BP fall), non-dippers (<10%), extreme dippers (>20%), and reverse dippers (rise at night). For clarity, systolic (or diastolic) pressure load was defined as the proportion of valid SBP (or DBP) readings above the prespecified threshold over the 24-h, daytime, or nighttime period, and dipping pattern was defined as the percent decline in mean nighttime relative to daytime BP (7, 10, 15).

### Echocardiographic assessment

Left ventricular mass was assessed via three-dimensional transthoracic echocardiography (Philips EPIQ CVx) performed by an experienced cardiologist blinded to ABPM results, following ASE/EACVI guidance (16, 17). LVH was defined as LVMI >115 g/m^2^ (men) and >95 g/m^2^ (women) using guideline criteria (16). Additional parameters included left ventricular ejection fraction (biplane Simpson), left atrial volume index, E/e′ ratio, and relative wall thickness (17).

### Laboratory analyses

Fasting serum samples assessed glucose, lipid profile, and serum creatinine. eGFR was calculated using the CKD-EPI 2021 equation (18).

### Statistical analysis

Statistical analyses were performed using MedCalc® Statistical Software, version 23.3.7 (32-bit) (MedCalc Software Ltd., Ostend, Belgium).^1^ Normality of continuous variables was assessed using the Shapiro–Wilk test. Results are reported as mean ± SD for normally distributed variables or median (IQR) for non-normally distributed variables. Categorical variables are expressed as frequencies and percentages.

Group comparisons between participants with and without LVH were performed using the Student’s t-test or Mann–Whitney U test for continuous variables, and the Chi-square test or Fisher’s exact test for categorical variables. Exact *p*-values are reported and interpreted cautiously, emphasizing magnitude and precision rather than dichotomizing findings as “significant” or “non-significant” (19).

**Figure 1:**
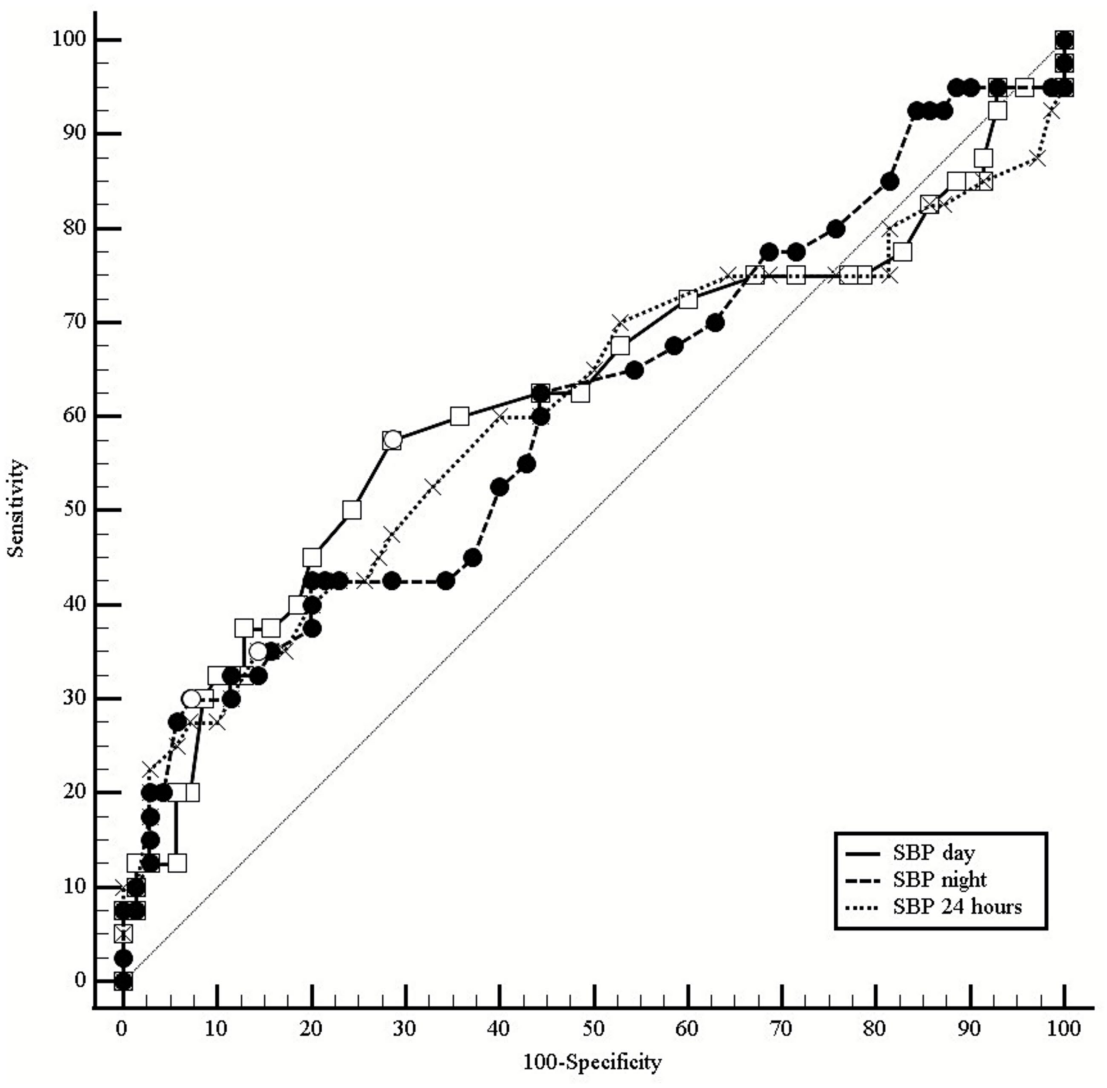
Comparative receiver operating characteristic (ROC) curves of ambulatory systolic blood pressure parameters for detection of left ventricular hypertrophy (LVH). Combined ROC curves comparing systolic BP parameters from 24-h ABPM for detecting echocardiographic LVH (*n* = 110; LVH prevalence 34.5%; LVMI >115 g/m^2^ men, >95 g/m^2^ women). Diagonal dashed line represents the reference line of no discrimination (AUC = 0.50). Results with 95% CI: Daytime SBP: AUC = 0.620 (0.523–0.717), *p* = 0.047. Nighttime SBP: AUC = 0.592 (0.493–0.691), *p* = 0.106. 24-h SBP: AUC = 0.591 (0.491–0.691), *p* = 0.126. Daytime SBP showed the best performance among systolic parameters, though all AUCs remained <0.65, indicating modest discriminative ability. Overlapping confidence intervals suggest no statistically significant differences between parameters. ROC, receiver operating characteristic; ABPM, ambulatory blood pressure monitoring; SBP, systolic blood pressure; LVH, left ventricular hypertrophy; LVMI, left ventricular mass index; AUC, area under the curve; CI, confidence interval.

The discriminative ability of ABPM parameters for detecting LVH was evaluated using receiver operating characteristic (ROC) curve analysis, calculating area under the curve (AUC) with 95% confidence intervals (CI), sensitivity, specificity, predictive values, likelihood ratios, and optimal cut-off points based on Youden’s index (20). Cutoff points derived from Youden’s index are presented as exploratory, data-driven thresholds and may be sample-dependent; external validation is required before clinical use as decision limits (20, 21). Diagnostic performance metrics are summarized in Table 1, and comparative ROC curves for systolic blood pressure parameters are illustrated in Figure 1.

**Table 1:** Diagnostic performance of ABPM parameters for detection of LVH using ROC analysis.

Parameter	AUC	95% CI	*p*-value	Cut-off	Sens (%)	Spec (%)	Youden’s J
Daytime SBP	0.620	0.523–0.717	0.047	≥134 mmHg	45.0	77.8	0.228
**Nighttime DBP**	**0.607**	0.508–0.706	**0.068**	≥69 mmHg	39.5	66.7	0.062
**Nighttime SBP**	**0.592**	0.493–0.691	**0.106**	≥119 mmHg	44.7	62.5	0.072
24-h SBP	0.591	0.491–0.691	0.126	≥129 mmHg	44.7	80.0	0.247
24-h DBP	0.520	0.419–0.621	0.730	≥79 mmHg	36.8	70.8	0.076
Daytime DBP	0.515	0.414–0.616	0.807	≥84 mmHg	34.2	73.6	0.078

*n* = 110; LVH prevalence 34.5%. LVH was defined as LVMI >115 g/m^2^ in men and >95 g/m^2^ in women (ASE/EACVI guidelines). AUC estimates are reported with 95% confidence intervals and exact *p*-values. Optimal cut-off points were derived using Youden’s index (J = sensitivity + specificity − 1). Parameters are ordered by descending AUC value. All AUCs were <0.65, indicating modest discriminative performance. Cut-off values are exploratory and sample-dependent; external validation is required before clinical application. ABPM, ambulatory blood pressure monitoring; AUC, area under the curve; CI, confidence interval; DBP, diastolic blood pressure; LVH, left ventricular hypertrophy; LVMI, left ventricular mass index; ROC, receiver operating characteristic; SBP, systolic blood pressure; Sens, sensitivity; Spec, specificity. Bold values indicate statistically significant results (*p* < 0.05).

Pre-specified subgroup analyses by sex, age category, antihypertensive treatment status, and blood pressure control were conducted on an exploratory basis to examine potential effect modification. However, given the modest overall sample size, the study was not powered for definitive subgroup comparisons; therefore, subgroup ROC results are not reported in detail (22, 23).

## Results

### Study population

**Figure 2:**
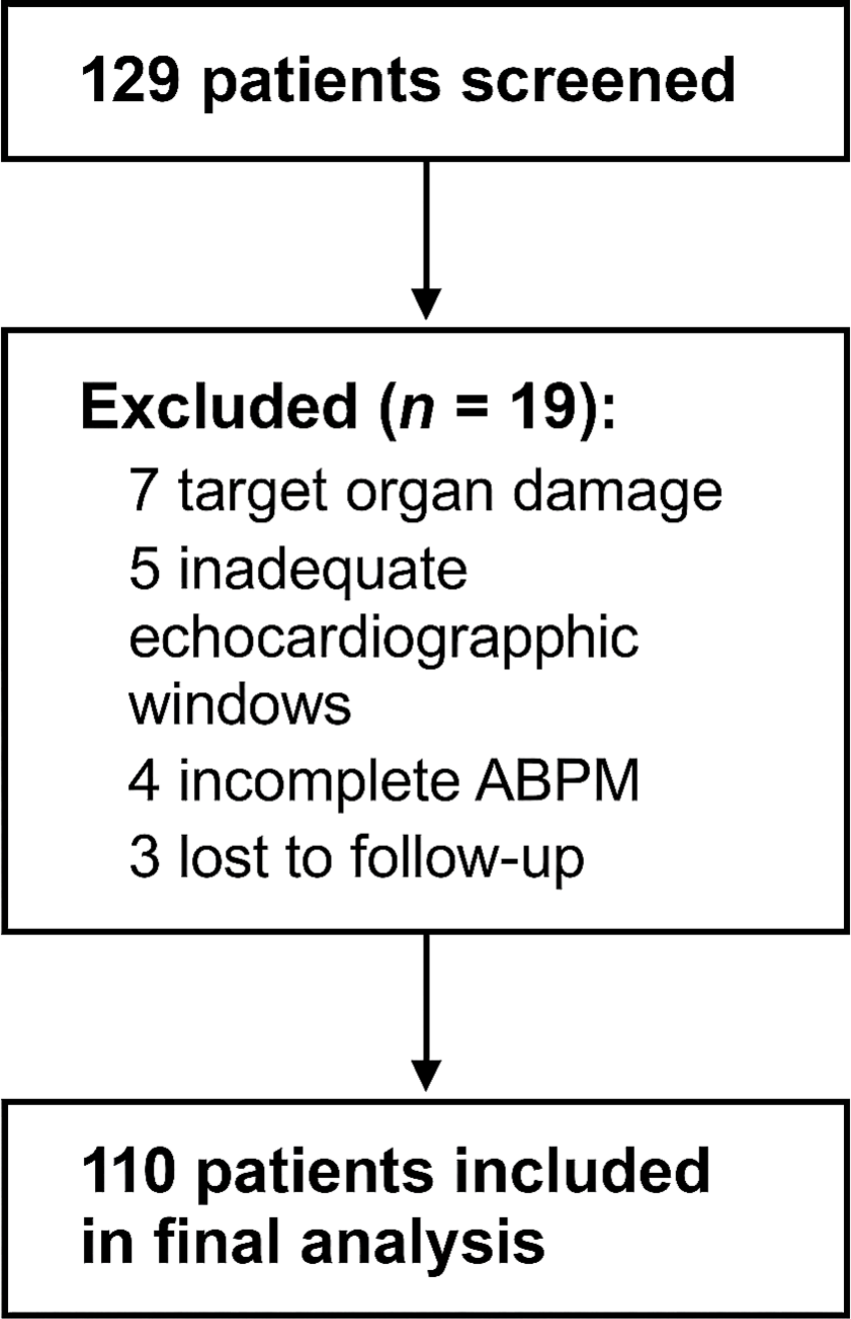
Flowchart of patient selection. Of 129 patients initially assessed for eligibility, 19 were excluded: 7 due to established extracardiac target organ damage, 5 with inadequate echocardiographic acoustic windows, 4 with incomplete ABPM recordings (<70% valid readings), and 3 lost to follow-up. The final analytic cohort comprised 110 patients. ABPM, ambulatory blood pressure monitoring.

A total of 129 patients were screened, of whom 19 were excluded: 7 due to established extracardiac target organ damage, 5 for inadequate echocardiographic acoustic windows, 4 for incomplete ABPM recordings, and 3 were lost to follow-up. The final analytic cohort comprised 110 participants (Figure 2).

### Baseline characteristics

The median age was 53 years (IQR: 42–63), and 56.4% were men. LVH was identified in 34.5% of participants (*n* = 38), consistent with prevalence rates reported in hypertensive cohorts (5). A prior diagnosis of hypertension was present in 63.6% of participants, with a median disease duration of 4 years (IQR: 1–10); duration was significantly longer among those with LVH compared to those without (8 vs. 3 years; *p* = 0.011). The majority of patients (87.3%) were receiving antihypertensive therapy at enrollment.

Cardiovascular risk factors were highly prevalent: alcohol consumption (42.7%), tobacco use (38.2%), sedentary lifestyle (52.7%), poor dietary habits (81.8%), and occupational stress (69.1%). Among women, 62.5% were postmenopausal. Type 2 diabetes mellitus was present in 10% of the overall cohort, with a trend toward higher prevalence in the LVH group (18.4% vs. 5.6%; *p* = 0.072). Median eGFR was 90.5 mL/min/1.73 m^2^ (IQR: 78.2–102.3), with no significant differences between groups. Table 2 summarizes baseline clinical and demographic characteristics.

**Table 2:** Baseline characteristics of the study population (*N* = 110).

Variable	Total (*N* = 110)	Normal LV mass (*n* = 72)	LVH (*n* = 38)	*p*-value
Age, years, median (IQR)	53 (42–63)	50.5 (40–62.3)	58 (50.5–69.8)	0.008ᵃ
Young adults (18–39)	13 (11.8)	11 (15.3)	2 (5.3)	
Adults (40–64)	72 (65.5)	48 (66.7)	24 (63.2)	
Older adults (≥65)	25 (22.7)	13 (18.1)	12 (31.6)	
Sex				0.400ᵇ
Female	48 (43.6)	34 (47.2)	14 (36.8)	
Male	62 (56.4)	38 (52.8)	24 (63.2)	
BMI, kg/m^2^, median (IQR)	28.9 (25.6–32.2)	28.8 (25.8–31.9)	29.4 (25.7–32.5)	0.682ᶜ
Underweight (<20)	1 (0.9)	1 (1.4)	–	
Normal (20–24.9)	23 (20.9)	15 (20.8)	8 (21.1)	
Overweight (25–29.9)	43 (39.1)	31 (43.1)	12 (31.6)	
Obesity I (30–34.9)	29 (26.4)	17 (23.6)	12 (31.6)	
Obesity II/III (≥35)	14 (12.7)	8 (11.1)	6 (15.8)	
Waist circumference, cm	98.0 (89–108)	97.0 (87–106)	99.5 (90.3–111)	0.099ᵃ
Normal	25 (22.7)	19 (26.4)	6 (15.8)	
High risk	18 (16.4)	11 (15.3)	7 (18.4)	
Very high risk	67 (60.9)	42 (58.3)	25 (65.8)	
Hypertension history				
Naïve (no treatment)	48 (43.6)	36 (50.0)	12 (31.6)	0.098ᵇ
Previous HTN diagnosis	70 (63.6)	43 (59.7)	27 (71.1)	0.334ᵇ
Duration, years	4 (1–10)	3 (1–7.5)	8 (3–12.5)	0.011ᵃ
≤5 years	42 (60.0)	30 (69.8)	12 (44.4)	
>5 years	28 (40.0)	13 (30.2)	15 (55.6)	
On treatment	62/70 (87.3)	36/43 (81.8)	26/27 (96.3)	0.139ᶜ
Risk factors				
Tobacco use				0.127ᶜ
Non-smoker	68 (61.8)	49 (68.1)	19 (50.0)	
Former smoker	31 (28.2)	18 (25.0)	13 (34.2)	
Current smoker	11 (10.0)	5 (6.9)	6 (15.8)	
Sedentarism	58 (52.7)	35 (48.6)	23 (60.5)	0.322ᵇ
Postmenopausal (women)	30/48 (62.5)	19/34 (55.9)	11/14 (78.6)	0.196ᶜ
Type 2 diabetes				0.072ᶜ
None	99 (90.0)	68 (94.4)	31 (81.6)	
<10 years	6 (5.5)	3 (4.2)	3 (7.9)	
10–20 years	4 (3.6)	1 (1.4)	3 (7.9)	
>20 years	1 (0.9)	–	1 (2.6)	
eGFR (MDRD), mL/min/1.73 m^2^	90.5 (71.7–107)	91.3 (73.3–106)	89.2 (69.4–106)	0.551ᶜ
≥90	59 (53.6)	40 (55.6)	19 (50.0)	
60–89	41 (37.3)	27 (37.5)	14 (36.8)	
30–59	10 (9.1)	5 (6.9)	5 (13.2)	

Data are presented as median (interquartile range [IQR]) for continuous variables and *n* (%) for categorical variables. LVH was defined as left ventricular mass index (LVMI) > 115 g/m^2^ in men and >95 g/m^2^ in women, according to ASE/EACVI guidelines. P-values were obtained using Mann–Whitney U test for continuous variables (a), Pearson chi-square test (b), or Fisher’s exact test (c). Exact *p*-values are reported and interpreted cautiously, emphasizing magnitude and precision. LVH, left ventricular hypertrophy; LVMI, left ventricular mass index; BMI, body mass index; WC, waist circumference; eGFR, estimated glomerular filtration rate; DM2, type 2 diabetes mellitus; IQR, interquartile range; ASE, American Society of Echocardiography; EACVI, European Association of Cardiovascular Imaging.

### ABPM parameters

Median systolic BP values were consistently higher in participants with LVH across all time periods: 24-h SBP (128 vs. 125 mmHg; *p* = 0.038), daytime SBP (133 vs. 130 mmHg; *p* = 0.043), and nighttime SBP (119 vs. 115 mmHg; *p* = 0.011). A dose–response pattern was observed, with LVH prevalence increasing across higher SBP strata: 80% among those with 24-h SBP ≥ 145 mmHg, 83.3% with daytime SBP ≥ 150 mmHg, and 72.7% with nighttime SBP ≥ 135 mmHg. In contrast, diastolic BP parameters showed no statistically significant associations with LVH status. Table 3 presents detailed ABPM findings stratified by LVH status.

**Table 3:** Ambulatory blood pressure monitoring (ABPM) parameters (mmHg).

Variable	Total (*N* = 110)	Normal LV mass (*n* = 72)	LVH (*n* = 38)	*p*-value
24-h SBP	126 (121–133)	125 (121–131)	128 (120–141)	0.038ᶜ
≤129	73 (66.4)	52 (72.2)	21 (55.3)	
130–134	12 (10.9)	8 (11.1)	4 (10.5)	
135–139	9 (8.2)	7 (9.7)	2 (5.3)	
140–144	11 (10.0)	4 (5.6)	7 (18.4)	
≥145	5 (4.5)	1 (1.4)	4 (10.5)	
24-h DBP	77.0 (72–82.8)	77.0 (71.8–82.3)	78.0 (72–82.8)	0.063ᶜ
Daytime SBP	131 (125–136)	130 (125–134)	133 (124–143)	0.043ᶜ
≤134	78 (70.9)	56 (77.8)	22 (57.9)	
135–139	10 (9.1)	6 (8.3)	4 (10.5)	
140–144	9 (8.2)	4 (5.6)	5 (13.2)	
145–149	7 (6.4)	5 (6.9)	2 (5.3)	
≥150	6 (5.5)	1 (1.4)	5 (13.2)	
Daytime DBP	81 (74–87)	81 (75–86.3)	79 (73–87)	0.261ᶜ
Nighttime SBP	117 (110–127)	115 (110–122)	119 (112–133)	0.011ᶜ
≤119	66 (60.0)	45 (62.5)	21 (55.3)	
120–124	13 (11.8)	12 (16.7)	1 (2.6)	
125–129	10 (9.1)	7 (9.7)	3 (7.9)	
130–134	10 (9.1)	5 (6.9)	5 (13.2)	
≥135	11 (10.0)	3 (4.2)	8 (21.1)	
Nighttime DBP	69 (64–75)	68 (64–73.5)	71 (64.5–75)	0.011ᶜ

Values are expressed as median (interquartile range [IQR]) for continuous variables or *n* (%) for categorical variables. LVH was defined as LVMI >115 g/m^2^ in men and >95 g/m^2^ in women. ABPM was performed using a validated oscillometric device with readings every 20 min (daytime: 06:00–22:00) and every 30 min (nighttime: 22:00–06:00). Statistical tests: ᵃMann–Whitney U test for continuous variables; ᵇPearson’s chi-square test; ᶜFisher’s exact test for categorical variables. Exact *p*-values are reported. ABPM, ambulatory blood pressure monitoring; SBP, systolic blood pressure; DBP, diastolic blood pressure; LVH, left ventricular hypertrophy; LVMI, left ventricular mass index; IQR, interquartile range.

### Diagnostic discrimination

ROC analysis revealed limited discriminative performance for LVH detection across all ABPM parameters, with all AUC values below 0.65. Among systolic parameters, daytime SBP demonstrated the highest discriminative ability (AUC = 0.620; 95% CI: 0.523–0.717; *p* = 0.047), followed by nighttime SBP (AUC = 0.592; 95% CI: 0.493–0.691; *p* = 0.106) and 24-h SBP (AUC = 0.591; 95% CI: 0.491–0.691; *p* = 0.126). Comparative ROC curves for these systolic parameters are illustrated in Figure 1.

Among diastolic parameters, nighttime DBP showed the best performance (AUC = 0.607; 95% CI: 0.508–0.706; *p* = 0.068), whereas 24-h DBP (AUC = 0.520; 95% CI: 0.419–0.621; *p* = 0.730) and daytime DBP (AUC = 0.515; 95% CI: 0.414–0.616; *p* = 0.807) demonstrated negligible discriminative ability.

Exploratory analysis of pressure load thresholds revealed that nighttime diastolic load >30% yielded high sensitivity (89.5%) but low specificity (29.2%; Youden’s J = 0.19), suggesting potential utility as a triage tool for ruling out very low-risk cases rather than for diagnostic confirmation. This threshold was derived from categorical analysis of pressure load (proportion of nocturnal readings exceeding DBP ≥ 70 mmHg) and represents a complementary approach to the continuous BP parameters evaluated in Table 1. As with all exploratory thresholds, external validation is required before clinical application (20, 21).

Pre-specified subgroup analyses by sex, age, treatment status, and BP control were explored; however, results are not reported in detail due to small strata sizes yielding imprecise ROC estimates with wide, overlapping confidence intervals that precluded reliable interpretation (22, 23).

## Discussion

This study evaluated the cross-sectional diagnostic discrimination of ABPM-derived indices for detecting echocardiographic LVH in a hypertensive cohort from coastal Ecuador. ABPM-derived parameters showed modest discrimination (all AUCs <0.65), comparable to prior reports in which ambulatory or office BP measures and ECG-based LVH criteria yield only low-to-moderate accuracy against echocardiography (24, 25). Such performance is insufficient for stand-alone screening or diagnosis and does not justify replacing echocardiography (17, 26). Accordingly, ABPM indices should be interpreted as complementary tools for risk stratification and for prioritizing imaging when echocardiography capacity is limited, rather than as diagnostic substitutes (26, 27). In particular, daytime systolic BP thresholds that provide a reasonable balance of sensitivity and specificity may support triage for echocardiographic evaluation, whereas nocturnal indices that may favor sensitivity over specificity in some settings may be more useful for identifying very low-risk cases rather than confirming LVH. Given the multifactorial determinants of LVH, single BP metrics are expected to have limited discriminative capacity, whereas multivariable models integrating ABPM with age, adiposity, metabolic factors, and hypertension duration typically achieve higher discrimination (25, 28).

Statistical significance alone does not establish clinical utility. Diagnostic usefulness should be judged by discrimination (AUC magnitude), classification performance (e.g., sensitivity/specificity and likelihood ratios at clinically relevant thresholds), and potential impact on care pathways rather than *p*-values alone (19, 21).

The 34.5% prevalence of LVH aligns with reports indicating that LVH affects roughly 30–40% of hypertensive populations and underscores the burden of hypertension-related organ damage (5). The association between older age and LVH is consistent with mechanisms linking cumulative pressure exposure, vascular remodeling, and myocardial hypertrophy (29).

Although international evidence consistently links elevated nocturnal BP and abnormal dipping patterns to LV hypertrophy and adverse cardiovascular outcomes, nocturnal ABPM parameters showed only modest or non-significant cross-sectional discrimination in our cohort (8, 9, 30). This may reflect limited power in a single-center cross-sectional study, the high prevalence of antihypertensive treatment (87.3%) with potential chronotherapy-related attenuation of nocturnal gradients, and possible misclassification introduced by fixed clock-time definitions of nighttime rather than individualized sleep periods (31, 32). In addition, our referral/high-risk case mix may modify effect sizes relative to population-based cohorts. These findings therefore do not refute prior literature; instead, they suggest that in treated and heterogeneous populations, nocturnal indices may have variable discriminatory performance for LVH and may be most informative when interpreted within multivariable models and evaluated in longitudinal studies assessing incremental value (30, 33).

### Strengths

Key strengths include the focus on a Latin American hypertensive cohort, the use of three-dimensional echocardiography as the reference standard for LVH assessment (supporting improved reproducibility in LV mass estimation compared with conventional approaches and aligning with ASE/EACVI guideline recommendations), and the comprehensive evaluation of daytime and nighttime ambulatory BP indices, supporting a detailed characterization of circadian BP patterns in this setting (10, 16, 17, 26).

### Clinical and policy implications

In resource-constrained settings, ABPM should be prioritized for patients with suspected white-coat or masked hypertension, marked discordance between office BP and overall cardiovascular risk, or high risk of hypertension-mediated organ damage (including suspected LVH), consistent with guideline-based indications (6, 13). When echocardiography capacity is limited, a daytime SBP threshold around 134 mmHg may serve as a pragmatic triage criterion to prioritize imaging referral in hypertensive patients; this value should not be interpreted as a diagnostic cutoff for LVH and requires external validation (21, 27).

To maximize the clinical utility of ABPM, primary care implementation should include standardized training (appropriate cuff selection and placement, patient instructions and diary use, minimum quality criteria for valid readings and day/night distribution, and guideline-based interpretation) (14). At the health-system level, expanding access to validated ABPM devices within primary care networks, integrating ABPM into standardized hypertension evaluation pathways for high-risk patients, and adopting risk-based referral algorithms combining clinical risk factors with ABPM findings may improve targeting of echocardiography and specialist care (10, 14). Regional multicenter studies with harmonized ABPM and echocardiography protocols are needed to validate thresholds and develop integrated prediction models for HMOD in Latin America (3, 10).

Scientific and Societal Implications.

Our findings support the use of ABPM as a complementary tool to characterize circadian BP patterns and to support risk stratification in hypertensive patients in resource-constrained settings. Given modest discrimination, ABPM indices should not replace echocardiography for LVH diagnosis; rather, they may help prioritize referral for imaging when capacity is limited. Broader implementation requires standardized training and quality criteria for ABPM acquisition and interpretation, and multicenter regional validation to ensure that any proposed thresholds are appropriate for diverse Latin American healthcare contexts (10, 13, 21).

### Limitations

This study was conducted in a single tertiary cardiovascular center with a modest sample size (*n* = 110). These design features may limit the precision of our estimates, increase the likelihood of type II error, and introduce referral-related selection bias. Accordingly, our results should be interpreted as exploratory and primarily applicable to urban, referred hypertensive adults in coastal Ecuador rather than to the broader and heterogeneous Latin American population, where ancestry, comorbidity burden, treatment patterns, and healthcare access differ across countries and levels of care. Larger multicenter studies across diverse Latin American settings (including primary, secondary, and tertiary care, as well as urban and rural populations) are needed to validate the ABPM cutoff values and diagnostic performance for LVH suggested by our data (10, 13, 26).

Because of the cross-sectional design, ABPM parameters and LVH were assessed at a single time point. This design permits only the identification of associations and does not allow robust causal inference, determination of temporal directionality, or evaluation of LVH progression/regression or long-term prognosis. Reverse causation, residual confounding, and selection/referral bias cannot be excluded; therefore, predictive or etiologic interpretations should be avoided (11, 12). Prospective cohort studies and treatment-response designs are required to determine whether ABPM-derived indices and thresholds predict incident LVH and whether changes in ambulatory BP are associated with LV mass regression over follow-up (30, 33).

Several exclusion criteria were applied to enhance the internal validity of ABPM and echocardiographic measurements. Patients with clinically significant arrhythmias were excluded because irregular RR intervals and frequent ectopy can impair the accuracy and reproducibility of oscillometric blood pressure measurement and increase the variability of ambulatory indices, as highlighted in contemporary guidance on blood pressure measurement and device validation (14, 34). Likewise, patients with advanced chronic kidney disease or established extracardiac hypertension-mediated organ damage were not included because CKD and end-stage kidney disease are strong, independent determinants of LV hypertrophy and abnormal LV geometry (e.g., through volume overload, anemia, and neurohormonal activation). However, these exclusions reduce the representativeness of our cohort, since multimorbidity, CKD, and overt cardiovascular disease are common among hypertensive patients in routine practice, including in Latin American populations. Our findings should therefore be interpreted as applicable primarily to relatively low-to-moderate risk ambulatory hypertensive adults, and future studies including patients with arrhythmias, CKD, and clinical HMOD—using validated measurement protocols for irregular rhythms—are needed to evaluate external validity of the diagnostic performance and exploratory ABPM thresholds suggested by our data (13, 14).

The study was not powered for robust subgroup analyses. Although stratified analyses by sex, age, antihypertensive treatment status, and blood pressure control were pre-specified, small numbers in several strata resulted in unstable and imprecise estimates. In line with methodological guidance on subgroup reporting, we therefore did not present detailed subgroup results and identify heterogeneity assessment as a priority for future, adequately powered multicenter studies (22, 23, 35).

Because diagnostic discrimination was modest (AUCs generally <0.65), individual ABPM parameters should not be used as stand-alone tests to diagnose or exclude LVH. Limited sensitivity for some thresholds implies a risk of false-negative classification; therefore, echocardiography remains necessary for definitive LVH assessment, and ABPM should be positioned as a complementary tool for risk stratification and triage rather than a replacement for imaging (21, 24).

### Future directions

Future studies should evaluate multivariable prediction models integrating ABPM parameters with clinical factors (e.g., age, sex, antihypertensive treatment, metabolic comorbidities, and hypertension duration) to assess whether discrimination improves beyond single BP-derived indices (25, 28). Prospective multicenter studies are needed to validate ABPM thresholds and assess whether ABPM indices predict incident LVH or longitudinal changes in LV mass (30, 33).

## Conclusion

In this Ecuadorian cohort, daytime systolic blood pressure with a threshold of ≥134 mmHg demonstrated the best diagnostic balance for identifying LVH, though overall discrimination remained modest. Nighttime systolic BP and other systolic parameters showed modest and often non-significant performance, while nocturnal diastolic load >30% provided high sensitivity but limited specificity (Youden’s J = 0.19), supporting its potential role primarily as a rule-out/triage marker rather than a confirmatory test.

These findings should be interpreted as hypothesis-generating. The proposed ABPM thresholds are exploratory and require external validation, ideally in larger prospective studies designed to evaluate incident LVH and longitudinal changes in LV mass (21, 27). From an implementation perspective, ABPM should be targeted to high-yield clinical scenarios (white-coat/masked hypertension and suspected HMOD) and embedded within standardized care pathways and training programs to support scalable adoption in Latin American health systems (10, 14). Expanding access to ABPM technology and standardized training may support earlier identification of HMOD and more efficient referral to echocardiography in resource-limited Latin American settings (3, 10).

## Data Availability

The raw data supporting the conclusions of this article will be made available by the authors, without undue reservation.

## Footnotes

1
www.medcalc.org
